# Characteristics and outcomes of adult patients hospitalised with RSV infection, influenza or COVID-19 in Belgium, winter seasons 2023/24 and 2024/25

**DOI:** 10.2807/1560-7917.ES.2026.31.37.2600088

**Published:** 2026-09-17

**Authors:** Yves Lafort, Cato Dambre, Yinthe Dockx, Anna Parys, Xavier Holemans, Marie-Pierre Parsy, Nicolas Dauby, Marijke Reynders, Marc Bourgeois, Arne Witdouck, Isabel Leroux-Roels, Eva Bernaert, Koen Magerman, Laura Cornelissen, Laurane De Mot

**Affiliations:** 1 Epidemiology of Infectious Diseases - Epidemiology and Public Health, Sciensano, Brussels, Belgium; 2 National Reference Centre for Respiratory Pathogens, Service of Viral Diseases, Sciensano, Brussels, Belgium; 3 Department of internal medicine and infectious diseases, Grand Hôpital de Charleroi – Les Viviers, Charleroi, Belgium; 4 Department of Infectious Diseases, Centre Hospitalier de Wallonie Picarde, Tournai, Belgium; 5 Department of Infectious Diseases, CHU Saint-Pierre; School of Public Health - Université Libre de Bruxelles (ULB); 6 AZ Sint-Jan Brugge, Laboratory Medicine, Molecular Microbiology, Bruges, Belgium; 7 Infectious Diseases Department, CHU UCL Namur, Yvoir, Belgium; 8 Department of Internal Medicine and Infectiology, Vitality Research Group (MIPI), Universitair Ziekenhuis Brussel, Vrije Universiteit Brussel (VUB), Jette, Belgium; 9 Department of Infection Control, Ghent University Hospital, Ghent, Belgium; Center for Vaccinology (CEVAC), Ghent University and Ghent University Hospital, Ghent, Belgium; 10 Department of Infection Prevention and Control, Ziekenhuis aan de Stroom, Antwerp, Belgium; 11 Department of Laboratory Medicine, Jessa Hospital, Hasselt, Belgium

**Keywords:** Respiratory Tract Infections, Respiratory Syncytial Viruses, Human Influenza, COVID-19, Patient Acuity, Belgium

## Abstract

**BACKGROUND:**

Severe acute respiratory infections (SARI) cause substantial disease burden in older adults and those with comorbidities.

**AIM:**

To inform Belgian vaccination policies, we aimed to compare risk profiles and outcomes of influenza, COVID-19 and RSV infections and examine age-related differences.

**METHODS:**

Adults (≥ 18 years) hospitalised with SARI in nine Belgian sentinel hospitals during the 2023/24 and 2024/25 winter seasons were included. Patients with laboratory-confirmed RSV infection, influenza or COVID-19 were compared in terms of demographics, comorbidities, complications and outcomes. Adults 18–64 years were compared with those ≥ 65 years. Multivariate analyses were applied.

**RESULTS:**

Among 2,806 patients, 535 (19.1%) had RSV infection, 1,694 (60.4%) influenza and 577 (20.6%) COVID-19. Patients with influenza were significantly younger (mean age 68.9 years) and had significantly fewer comorbidities (89.7%) than patients with RSV (73.8 years; 95.8%) or COVID-19 (74.0 years; 93.6%). Patients with COVID-19 experienced significantly fewer complications (40.8%) than patients with RSV (48.9%) or influenza (45.2%). Patients with RSV had higher in-hospital mortality (11.9%) than patients with influenza (7.3%; p = 0.089) or COVID-19 (6.9%; p = 0.037). Across pathogens, younger adults had relatively more comorbidities after adjusting for differences in the general population. They had shorter hospital stays and lower mortality, but higher intensive care unit admission rates.

**CONCLUSION:**

Patients with RSV infection had a disease course comparable to patients with influenza or COVID-19 and a higher mortality rate, especially with increasing age. These findings highlight the importance of RSV vaccination in older adults and those with comorbidities, alongside established vaccination strategies for seasonal influenza and COVID-19.

**Key public health message**

**What did you want to address in this study and why?**

In 2025, reimbursement of RSV vaccination of adults ≥75 years and adults ≥60 years with certain comorbidities was approved in Belgium. There is a need to better understand the risk factors and disease progression in adults hospitalised with RSV compared with the other major viral respiratory pathogens influenza virus and SARS-CoV-2. To address this, we analysed data from the hospital sentinel surveillance system, which provides surveillance data from hospitals across Belgium.

**What have we learnt from this study?**

The risk profile of hospitalised adult patients infected with RSV – in terms of age and underlying conditions – largely corresponds to that of patients hospitalised with influenza or COVID-19. The course of the disease in hospitalised adult RSV patients is as severe as influenza and COVID-19, and in the past two seasons hospitalised RSV patients have experienced even more complications and the mortality rate was higher.

**What are the implications of your findings for public health?**

Each year, a substantial proportion of at-risk adults in Belgium are vaccinated against influenza and COVID-19; however, this is not yet the case for RSV. Our findings can help inform a vaccination strategy for RSV.

## Introduction

Each year, severe acute respiratory infections (SARI) cause substantial morbidity and mortality among adults 65 years and older and individuals with comorbidities [1,2], putting pressure on health systems. Influenza virus has long been a leading viral cause of SARI, and since 2020 severe acute respiratory syndrome coronavirus 2 (SARS-CoV-2) has become another major pathogen. Respiratory syncytial virus (RSV) is also recognised as an important cause of severe illness in older and immunocompromised adults [3,4]. Susceptibility to these pathogens increases with age because of multimorbidity, physiological decline and immunosenescence. Furthermore, atypical clinical presentations in older adults frequently delay diagnosis and treatment leading to prolonged hospital stays and increased complications and mortality [2].

In Belgium, annual influenza vaccination has been available for more than 15 years for high-risk adults, including individuals aged 65 years or older, nursing home residents and those with chronic conditions affecting the lungs, heart (excluding hypertension), liver or kidneys, as well as patients with metabolic, neuromuscular or immune disorders or a body mass index (BMI) greater than or equal to 40 kg/m^2^ [5]. Since the COVID-19 pandemic, vaccination against COVID-19 is recommended for high-risk adults using similar criteria to the influenza vaccination, but also including hypertension, neurological and cognitive disorders and selected rare diseases [6]. However, COVID-19 vaccination remains free for the entire population.

In 2023, two RSV vaccines were licensed in Belgium. In December 2024, the Superior Health Council (SHC) recommended RSV vaccination for adults 75 years or older, adults 60 years or older with risk factors, immunodeficient individuals and nursing home residents [7]. Reimbursement became available in August 2025 but only for adults 65 years or older in residential care or with chronic respiratory disease, chronic heart failure, chronic kidney disease, diabetes, obesity (BMI ≥ 30 kg/m^2^) or immunodeficiency [8].

To better inform these recommendations, we conducted a comparative analysis of risk factors and disease progression among adults hospitalised with SARI caused by influenza virus, SARS-CoV-2 or RSV.

## Methods

### Study setting and data collection

We conducted a retrospective cohort study of adults (≥ 18 years) hospitalised with SARI who tested positive for influenza virus, RSV or SARS-CoV-2. Coinfections were excluded.

Data were retrieved from the Belgian SARI surveillance network, coordinated by the Belgian National Public Health Institute (Sciensano) [9], which includes nine hospitals (10 until November 2024) and prospectively monitors all hospitalised SARI patients from admission until discharge. The network covers patients from the three Belgian regions and all participating hospitals have large intensive care units. They account for ca 20% of all accredited hospital beds in Belgium. We analysed two winter seasons: 2023/24 (13 November 2023–30 April 2024) and 2024/25 (1 September 2024–30 April 2025). Patients admitted before 13 November 2023 were excluded because of major changes to the surveillance (digital reporting, expanded participation, questionnaire revision, improved completeness and a new SARI case definition) introduced on that date.

Participating hospitals collected demographic, clinical (symptoms, comorbidities, treatments and complications) and outcome information from patient’s medical records. Data were submitted weekly by batch upload or via an online questionnaire and compiled into an R-based database [10].

### Severe acute respiratory infection case definition

Severe acute respiratory infection cases were defined as follows: (i) hospitalisation ≥ 24 hours; (ii) at least two acute respiratory symptoms (fever ≥ 38 °C or history of fever, cough, respiratory distress such as dyspnoea or abnormal lung auscultation); (iii) symptom onset within 10 days before admission; and (iv) exclusion of nosocomial infections (symptom onset > 24 h after admission).

### Testing for respiratory viruses

Respiratory specimens (nose/throat swabs (90.5%), nasopharyngeal aspirates (9%) or bronchoalveolar lavage specimens (0.5%)) were obtained from all patients meeting the SARI case definition and tested at the National Reference Centre for Respiratory Pathogens (NRC). Viral nucleic acids were extracted with the eMAG system (bioMérieux, France). Samples were tested using commercial or in-house multiplex qPCR assays for influenza virus, SARS-CoV-2 and RSV [9].

### Statistical analysis

First, we conducted descriptive comparisons of sociodemographic characteristics, comorbidities, treatments, complications and outcomes across infections. Patients with missing data for a given variable were systematically excluded from the corresponding analysis. Second, we stratified analyses by age (18–64 years vs ≥ 65 years, and further, within older adults 65–74, 75–84 and ≥ 85 years). Where age-specific prevalence estimates for comorbidities in the general population were available, we calculated a weighted prevalence (weighted PR) by dividing the ratio of the comorbidity prevalence among SARI patients 65 years or older over the prevalence among SARI patients 18–64 years by the corresponding ratio of the prevalence in the general population. We also calculated the ratio of the prevalence among SARI patients over the prevalence in the general population (ratio SARI/general population). We used the results of the Sciensano Health Interview Survey 2023–2024 for this purpose [11].

Statistical testing used t-tests or Wilcoxon rank-sum tests for continuous variables, chi-square tests for categorical variables and Fisher’s exact tests for binary variables. Multivariable logistic regression models assessed pathogen-specific differences, adjusting for sex, age and nursing home residence when analysing comorbidities and additionally for the presence of at least one comorbidity when analysing treatments, complications and outcomes. Antiviral treatment was not associated with disease course and therefore not included in the model. Adjusted odds ratios (aOR) with 95% confidence intervals (CI) and p values were calculated, with p < 0.05 considered statistically significant. Analyses were conducted using Stata version 17 (StataCorp, College Station, United States (US)).

## Results

### Incidence of hospital admissions

The Figure shows the weekly incidence of hospital admissions due to RSV infection, influenza or COVID-19 over the study period. In the 2023/24 season, there were overlapping epidemics of the three infections. In the 2024/25 season, the RSV and influenza epidemics overlapped, but COVID-19 occurred mainly outside the winter period.

**Figure:**
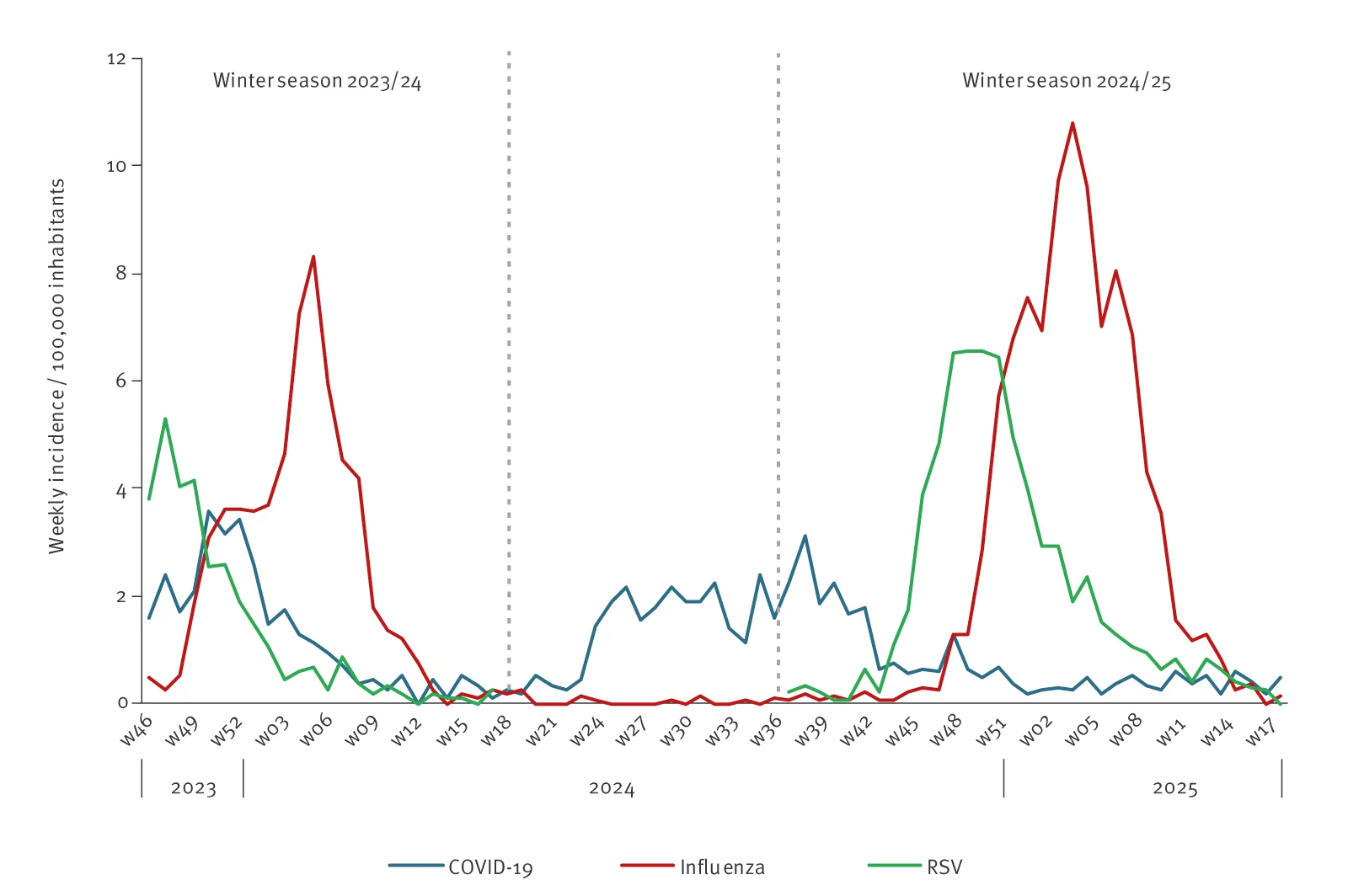
Weekly incidence of hospital admissions with severe acute respiratory infection, by infection, Belgium, 2023–2025

RSV: respiratory syncytial virus; w: week.

### Sociodemographic characteristics

Across both seasons studied, 9,085 adult patients were hospitalised with SARI, of whom 9,064 (99.8%) had a test result available for the three pathogens. Of these, 2,863 (31.6%) tested positive for at least one of the three viruses. After exclusion of 57 patients coinfected with at least two viruses, 2,806 patients were included, 535 with RSV infection (19%), 1,694 with influenza (60%), and 577 with COVID-19 (21%). The mean age of influenza patients (68.9 years) was significantly lower than that of patients with RSV (73.8 years; p < 0.001) and COVID-19 (74.0 years; p < 0.001) (Table 1). A higher proportion of patients with COVID-19 were male (56.0%) compared with RSV patients (50.1%; p = 0.049). Patients with RSV were more likely to reside in nursing homes (18.3%) than patients with influenza (10.0%; p < 0.001) or COVID-19 (11.4%; p = 0.002).

**Table 1:** Sociodemographic characteristics and risk factors in adult patients hospitalised for respiratory syncytial virus infection, influenza and COVID-19, by infection, Belgium, winter seasons 2023/24 (13 November 2023–30 April 2024) and 2024/25 (1 September 2024–30 April 2025)

Characteristics	RSV (n = 535) %	Influenza (n = 1,694) %	COVID-19 (n = 577) %	RSV vs Influenza			RSV vs COVID-19			Influenza vs COVID-19		
				p value			p value			p value		
Age in years, mean (SD)	73.8 (14.9)	68.9 (17.3)	74.0 (14.8)	< 0.001			0.797			< 0.001		
Age in years, median (Q1–Q3)	76 (65–86)	72 (60–81)	77 (67–84)	< 0.001			0.967			< 0.001		
Male	50.1	53.6	56.0	0.157			0.049			0.322		
Risk factors				aOR^a^	95% CI	p value	aOR^a^	95% CI	p value	aOR^a^	95% CI	p value
Nursing home resident	18.3	10.0	11.4	1.56	1.13 to 12.16	**0.007**	1.76	1.20 to 12.58	**0.004**	1.16	0.83 to 11.64	0.380
At least one comorbidity	95.8	89.7	93.6	1.98	1.23 to 13.17	**0.005**	1.29	0.73 to 12.29	0.378	0.64	0.42 to 10.96	**0.032**
Asthma	15.5	12.3	9.5	1.47	1.09 to 11.20	**0.012**	1.80	1.22 to 12.66	**0.003**	1.19	0.84 to 11.67	0.323
Other chronic respiratory disease	34.8	30.5	33.9	1.26	1.01 to 11.58	**0.041**	1.14	0.88 to 11.49	0.327	0.92	0.74 to 11.14	0.424
Chronic cardiac disease	48.0	36.9	45.8	1.38	1.10 to 11.73	**0.005**	1.23	0.94 to 11.60	0.137	0.89	0.72 to 11.11	0.310
Hypertension	41.6	37.6	35.6	0.99	0.79 to 11.24	0.908	1.29	0.99 to 11.69	0.060	1.31	1.06 to 11.64	**0.014**
Diabetes	24.6	23.9	21.5	0.94	0.73 to 11.20	0.610	1.15	0.85 to 11.56	0.363	1.23	0.96 to 11.57	0.105
Obesity	10.0	13.1	10.7	0.71	0.50 to 11.01	0.059	0.87	0.57 to 11.31	0.501	1.26	0.91 to 11.73	0.163
Liver insufficiency	4.5	3.8	4.7	1.13	0.67 to 11.92	0.647	1.00	0.54 to 11.86	0.996	0.86	0.52 to 11.43	0.560
Renal insufficiency	27.8	21.1	22.8	1.17	0.92 to 11.51	0.206	1.35	1.00 to 11.82	0.052	1.14	0.89 to 11.47	0.298
Immunodeficiency	15.5	12.7	17.9	1.36	1.01 to 11.84	**0.045**	0.92	0.65 to 11.30	0.636	0.67	0.51 to 10.90	**0.007**
Organ transplant	1.5	1.8	2.0	1.11	0.47 to 12.62	0.815	0.72	0.28 to 11.89	0.504	0.66	0.32 to 11.39	0.277
Stem cell transplant	2.8	1.9	1.6	1.82	0.89 to 13.73	0.214	1.74	0.70 to 14.32	0.236	0.99	0.44 to 12.25	0.984
Solid cancer	23.1	17.6	21.4	1.26	0.97 to 11.64	0.084	1.18	0.87 to 11.60	0.299	0.95	0.73 to 11.23	0.686
Haematologic cancer	12.2	6.8	11.2	2.02	1.42 to 12.89	**< 0.001**	1.19	0.80 to 11.78	0.387	0.60	0.42 to 10.85	**0.004**
Other haematologic disease	4.1	4.8	8.0	0.70	0.31 to 11.57	0.386	0.51	0.22 to 11.19	0.117	0.69	0.38 to 11.25	0.220
Neurologic disease	14.3	12.6	14.8	1.01	0.73 to 11.40	0.928	0.87	0.60 to 11.27	0.471	0.85	0.63 to 11.15	0.288
Neuromuscular disease	3.1	2.9	5.8	1.08	0.57 to 12.02	0.821	0.43	0.22 to 10.83	**0.012**	0.42	0.26 to 10.69	**0.001**
Cognitive disturbance	14.3	13.3	13.0	0.75	0.54 to 11.04	0.086	0.95	0.64 to 11.41	0.801	1.26	0.91 to 11.73	0.158

aOR: adjusted odds ratio; CI: confidence interval; Q: quartile; RSV: respiratory syncytial virus; SD: standard deviation.

Bold indicates statistically significant associations (p < 0.05).

^a^ Nursing home residence was adjusted for age and sex.

All comorbidities were adjusted for age, sex and nursing home residence.

### Risk factors

After adjusting for age and sex, nursing home residency remained significantly more prevalent among RSV patients. Patients with influenza were significantly less often having at least one comorbidity (89.7%) than patients with RSV (95.8%; p = 0.005) or COVID-19 (93.6%; p = 0.032). These differences remained after adjusting for sex, age and nursing home residency. Immunodeficiency (from any cause) and haematologic cancer (whether active or in remission) were significantly less prevalent among patients with influenza. After adjusting for immunodeficiency, haematologic cancer was still significantly less prevalent. However, immunodeficiency was no longer significantly less prevalent after adjusting for haematologic cancer (aOR RSV/influenza aOR: 1.085; p = 0.632. Influenza/COVID-19 aOR: 0.77; p = 0.101) suggesting the association with immunodeficiency may be explained by overlap with haematologic malignancies. Compared with RSV patients, influenza patients had significantly lower prevalence of chronic respiratory diseases (excluding asthma) and chronic cardiac disease. Compared with patients with COVID-19, influenza patients had a significant higher prevalence of hypertension. Asthma was significantly more prevalent among patients with RSV, while neuromuscular disease was significantly more prevalent among patients with COVID-19.

Adults aged 65 years or older were more frequently residents of nursing homes and had a higher prevalence of comorbidities, including chronic respiratory disease (other than asthma), chronic cardiac disease, hypertension, diabetes, renal insufficiency, cognitive impairment, solid cancer, non-malignant haematological disorders and neurological conditions (Table 2). Conversely, asthma, immunodeficiency, a history of solid organ transplantation, stem cell transplantation and neuromuscular disease were significantly more prevalent in patients aged 18–64 years. Nursing home residency and comorbidities found to be more common in SARI patients 65 years and older increased with advancing age in both age groups, while comorbidities found to be less common declined further (the Supplement presents sociodemographic characteristics and risk factors by the smaller age categories of 18–54, 55–64, 65–74, 75–84 and ≥ 85 years).

**Table 2:** Sociodemographic characteristics and underlying conditions of adult patients hospitalised for respiratory syncytial virus infection, influenza or COVID-19 and comparison with general population, by age category, Belgium, winter seasons 2023/24 (13 November 2023–30 April 2024) and 2024/25 (1 September 2024–30 April 2025)

Characteristics	Prevalence in general population		Prevalence in SARI patients						Ratio SARI/ general population	
	18–64 years %	≥ 65 years %	18–64 years (n = 798) %	≥ 65 years (n = 2,008) %	PR	95% CI	p value	Weighted PR	18–64 years	≥ 65 years
Sociodemographic characteristics										
Female	NA	NA	52.4	53.8	1.03	0.95 to 11.11	0.486	NA	NA	NA
Nursing home resident	NA	NA	1.9	16.0	8.57	4.95 to 114.84	**< 0.001**	NA	NA	NA
Underlying conditions										
At least one	NA	NA	81.6	95.8	1.17	1.13 to 11.22	**< 0.001**	NA	NA	NA
Asthma	6.5	6.1	16.3	10.7	0.65	0.53 to 10.81	**< 0.001**	0.7	2.5	1.8
Other chronic respiratory disease	2.5	6.9	27.9	33.7	1.21	1.06 to 11.38	**0.004**	0.4	11.2	4.9
Chronic cardiac disease	3.2	12.7	18.9	49.8	2.64	2.26 to 13.09	**< 0.001**	0.7	5.9	3.9
Hypertension	10.8	33.5	19.8	45.3	2.29	1.97 to 12.67	**< 0.001**	0.7	1.8	1.4
Diabetes	4.4	15.3	14.8	27.0	1.83	1.51 to 12.20	**< 0.001**	0.5	3.4	1.8
Obesity	16.5	18.8	11.2	12.3	1.10	0.86 to 11.40	0.450	1.0	0.7	0.7
Liver insufficiency	0.8	1.2	5.1	3.8	0.73	0.50 to 11.08	0.116	0.5	6.4	3.2
Renal insufficiency	2.3	5.8	11.2	27.4	2.44	1.97 to 13.02	**< 0.001**	1.0	4.9	4.7
Cognitive disturbance	0.4	4.8	5.1	16.7	3.29	2.37 to 14.55	**< 0.001**	0.3	12.8	3.5
Immunodeficiency	NA	NA	19.9	12.1	0.61	0.50 to 10.73	**< 0.001**	NA	NA	NA
History of organ transplant	NA	NA	3.6	1.1	0.31	0.17 to 10.55	**< 0.001**	NA	NA	NA
Stem cell transplant	NA	NA	3.6	1.4	0.41	0.23 to 10.72	**0.002**	NA	NA	NA
Solid cancer	NA	NA	11.8	22.5	1.91	1.54 to 12.37	**< 0.001**	NA	NA	NA
Haematologic cancer	NA	NA	8.9	8.6	0.97	0.74 to 11.27	0.823	NA	NA	NA
Other haematologic disease	NA	NA	2.8	6.5	2.29	1.25 to 14.20	**0.005**	NA	NA	NA
Neurologic disease	NA	NA	9.4	15.0	1.60	1.25 to 12.04	**< 0.001**	NA	NA	NA
Neuromuscular disease	NA	NA	4.7	3.1	0.66	0.44 to 11.00	**0.049**	NA	NA	NA

CI: confidence interval; NA: not applicable; PR: prevalence ratio; SARI: severe acute respiratory infection.

Bold indicates statistically significant associations (p < 0.05).

Weighted prevalence ratio was calculated by dividing the prevalence ratio for SARI patients aged ≥ 65 years vs those aged 18–64 years by the corresponding prevalence ratio in the general population.

Comorbidities, for which the age-specific prevalence in the general population was known, were several times more prevalent among SARI patients than in the general population for both 18–64-year-olds and those 65 years and older, except for obesity, which was less prevalent among SARI patients. The distribution of comorbidities generally showed the same pattern across the two age groups in both SARI patients and the general population, although the difference between the age groups was usually more pronounced in the general population. After accounting for the underlying population pattern by weighting the prevalence ratio of  ≥ 65-year-old SARI patients over 18–64-year-old patients by the prevalence ratio in the general population, the observed age-specific differences among SARI patients were largely resolved, indicating that relative to the general population, SARI patients aged 18–64 years had a higher prevalence of comorbidities than SARI patients 65 years and older.

### Hospitalisation outcome

Length of stay, excluding patients transferred to or from another hospital, did not significantly differ among the three pathogens, nor did ICU admission, length of ICU stay or invasive mechanical ventilation (Table 3). Patients with COVID-19 had a significantly lower complication rate (40.8%) than patients with RSV (48.9%; p = 0.010) or influenza (45.2%; p = 0.018). This was particularly the case for pneumonia and acute renal insufficiency. Differences in cardiovascular complications, acute respiratory distress syndrome and sepsis were too small to be significant. Other complications were too rare to show significant differences (data not shown). Patients with RSV had higher in-hospital mortality (11.9%) than patients with influenza (7.3%) or COVID-19 (6.9%). After adjusting for sex, age, nursing home residence and comorbidities, in-hospital mortality remained higher among patients with RSV than among patients with COVID-19 (aOR: 1.64; 95% CI: 1.03 to 12.61; p = 0.037), but the difference between patients with RSV and influenza was no longer statistically significant (aOR: 1.38; 95% CI: 0.95 to 12.00); p = 0.089).

**Table 3:** Treatments, complications and disease outcome in adult patients hospitalised for respiratory syncytial virus infection, influenza or COVID-19, by infection, Belgium, winter seasons 2023/24 (13 November 2023–30 April 2024) and 2024/25 (1 September 2024–30 April 2025)

Hospitalised patients	RSV (n = 535) %	Influenza (n = 1,694) %	COVID-19 (n = 577) %	RSV vs influenza			RSV vs COVID-19			Influenza vs COVID-19		
				aCoeff	95% CI	p value	aCoeff	95% CI	p value	aCoeff	95% CI	p value
Days of stay												
Days of stay^a^, median (Q1–Q3)	6 (4.0–12.0)	5 (3.0–10.0)	6 (4.0–11.0)	0.34	-0.38 to 1.06	0.353	0.47	-0.35 to 1.29	0.264	0.08	-0.54 to 0.69	0.806
Days of stay ICU, median (Q1–Q3)	7 (3.0–13.5)	5 (3.0–9.5)	7 (3.0–13.5)	-0.39	-2.44 to 1.65	0.705	-0.50	-3.21 to 2.21	0.716	< 0.01	-2.13 to 2.13	1.000
Treatment				aOR	95% CI	p value	aOR	95%CI	p value	aOR	95% CI	p value
ICU admission	12.9	14.5	10.9	0.98	0.71 to 1.34	0.882	1.18	0.80 to 11.73	0.408	1.20	0.88 to 11.64	0.254
Invasive mechanical ventilation	4.3	5.7	3.8	0.80	0.47 to 1.37	0.421	1.03	0.53 to 12.00	0.931	1.30	0.77 to 12.19	0.325
Complications (% yes)												
At least one complication	48.9	45.2	40.8	1.08	0.87 to 11.33	0.498	1.40	1.08 to 11.80	**0.010**	1.28	1.04 to 11.58	**0.018**
Pneumonia	32.2	29.2	25.1	1.17	0.93 to 11.46	0.181	1.50	1.14 to 11.98	**0.004**	1.27	1.01 to 11.59	**0.043**
Acute renal insufficiency	17.8	14.3	12.8	1.14	0.86 to 11.51	0.359	1.53	1.07 to 12.18	**0.019**	1.33	0.99 to 11.80	0.061
Cardiovascular	9.0	7.4	9.0	1.14	0.74 to 11.75	0.547	1.03	0.63 to 11.68	0.919	0.93	0.62 to 11.39	0.724
ARDS	6.3	6.0	5.2	1.02	0.67 to 11.56	0.918	1.25	0.74 to 12.12	0.408	1.23	0.74 to 12.06	0.363
Sepsis	5.4	3.8	3.1	1.24	0.68 to 12.28	0.483	1.40	0.66 to 12.99	0.379	1.19	0.62 to 12.26	0.604
Disease outcome^b^												
Cured	84.2	88.4	89.2	0.81	0.59 to 11.12	0.204	0.71	0.48 to 11.06	0.095	0.88	0.63 to 11.23	0.459
Deceased	11.9	7.3	6.9	1.38	0.95 to 12.00	0.089	1.64	1.03 to 12.61	**0.037**	0.81	0.59 to 11.12	0.204
Sequelae^c^	4.0	4.4	3.9	0.92	0.51 to 11.63	0.764	0.92	0.46 to 11.82	0.810	1.07	0.62 to 11.83	0.813

aCoeff: adjusted regression coefficient; aOR: ICU: intensive care unit; Q: quartile; RSV: respiratory syncytial virus.

^a^ Patients transferred in and patients transferred out excluded.

^b^ Patients transferred out and outcome unknown excluded.

^c^ A patient is considered as having sequelae when leaving the hospital with remaining symptoms with the expectation that they will be long lasting.

Bold indicates statistically significant associations (p < 0.05).

All variables adjusted for age, sex, nursing home residency and presence of at least one comorbidity.

Severe acute respiratory infection patients aged 65 years or older had significantly longer hospital stays than patients aged 18–64 years, and length of stay further increased with age among patients 65 years and older (Table 4). Admission to ICUs decreased significatively with age. No significant difference was observed in length of ICU stay between the < 65 and ≥ 65 years age groups, but length of ICU stay decreased significantly with increasing age among patients 65 years and older. The use of invasive mechanical ventilation also declined with age. Patients aged 65 years or older experienced complications more often than younger patients, although the difference was not statistically significant. Overall, the case fatality rate increased with age, although it was lower in 75–84-year-olds than in 65-74-year-olds. It increased from 3.4% among patients aged 18–64 years to 17.0% among patients 85 years and older. By infection, the case fatality rate increased from 3.4% to 21.5% among patients with RSV, from 3.9% to 17.2% among patients with influenza and from 0.9% to 12.0% among patients with COVID-19.

**Table 4:** Hospitalisation characteristics, complications and outcome in adult patients hospitalised for respiratory syncytial virus infection, influenza or COVID-19, by age category, Belgium, winter seasons 2023/24 (13 November 2023–30 April 2024) and 2024/25 (1 September 2024–30 April 2025)

Hospitalisation and outcome	18–64 years (n = 798) %	≥ 65 years (n = 2,008) %	p value	65–74 years (n = 654) %	75–84 years (n = 751) %	≥ 85 years (n = 603) %	p value
Days of stay^a^, median (Q1–Q3)	4 (3–8)	7 (4–12)	**< 0.001**	5 (3–10)	7 (4–11)	8 (5–13)	**< 0.001**
ICU admission	17.0	12.1	**< 0.001**	20.5	10.6	5.0	**< 0.001**
Days of stay ICU, median (Q1–Q3)	4 (2–10)	5 (3–9)	0.922	5 (3–10)	5 (2–9)	3.5 (3–5)	**0.014**
Invasive mechanical ventilation	7.4	4.0	**< 0.001**	7.2	4.0	0.4	**< 0.001**
At least one complication	42.3	46.1	0.075	44.9	46.6	46.9	0.762
Cured	92.4	85.8	**< 0.001**	86.2	90.1	79.6	**< 0.001**
Deceased	3.4	10.1	**< 0.001**	8.0	6.5	17.0	**< 0.001**
Sequelae^b^	4.2	4.2	0.976	5.9	3.4	3.4	0.052

ICU: intensive care unit; Q: quartile.

^a^ Patients transferred in and patients transferred out were excluded.

^b^ A patient is considered to have sequelae when leaving the hospital with symptoms that are expected to be long lasting.

Bold indicates statistically significant associations (p < 0.05).

## Discussion

This study is the first in Belgium to compare risk profiles and disease progression among adults hospitalised with SARI caused by infections with influenza virus, SARS-CoV-2 or RSV.

Overall, patient characteristics were similar across infections, although patients with influenza were generally younger and had fewer comorbidities than patients with RSV or COVID-19, consistent with most prior studies [12-16] including those assessing post-pandemic COVID-19 cases [15,16]. While some studies reported age differences between patients with RSV and COVID-19 [15], we did not observe this. The lower prevalence of comorbidities among influenza patients, even after adjusting for sex, age and nursing home residence, aligns with previous work [12,13,15,17-19], suggesting a greater role of age and multimorbidity in RSV and COVID-19 severity. Three prior studies reported a higher prevalence of comorbidities in patients with RSV than in those with COVID-19 [12,15,19]. We observed the same trend, although it was not statistically significant. Patients with RSV were also significantly more likely to reside in nursing homes, even after adjusting for age.

Across the three viral infections, the same underlying conditions were frequently reported, including cardiac, respiratory and renal comorbidities, as well as immune disorders, malignancies and diabetes. Overall risk profiles were therefore largely comparable, although most comorbidities were less prevalent among patients with influenza. Specifically, cancers, chronic cardiac disease, immunodeficiency and chronic respiratory disease other than asthma were less prevalent among patients with influenza. Asthma was more prevalent among patients with RSV, while neuromuscular disease was most prevalent among patients with COVID-19. The clinical relevance of these pathogen-specific differences remains uncertain, as they may reflect residual confounding or chance. Some prior studies reported similar findings, such as higher prevalence of asthma and renal insufficiency among patients with RSV [12,13,17], whereas others identified different patterns.

Comparison of comorbidity prevalence across age groups is challenging, as many comorbid conditions increase with age. To account for this, we adjusted comorbidity prevalence ratios in younger (18–64 years) and older (≥ 65 years) adults using age-specific reference data from the general population, where available. Before adjustment, comorbidities such as chronic cardiac disease, hypertension, diabetes and obesity appeared to be several times more common among older SARI patients than younger ones. However, after adjusting for age-related prevalence in the general population, the prevalence ratio was lower. This suggests that the higher prevalence of comorbidities in older SARI patients is largely attributable to age-related baseline rates rather than to an age-specific increase in risk due to comorbidities. Notably, the rate of comorbidities in SARI patients compared with the general population is higher among younger patients than older patients. This might suggest that for older adults, age itself is a more important risk factor than underlying health conditions, which makes the case for vaccinating healthy elderly people even stronger. Moreover, risk factors not inherently linked to ageing - such as immunodeficiency, organ transplantation and asthma - were relatively more prevalent among younger adults. The SARI-to-general population ratio further supports that comorbidities including asthma and other chronic respiratory diseases, chronic cardiac disease, diabetes, liver and renal disease and cognitive disorders are risk factors for SARI. Obesity (defined as BMI > 30 kg/m^2^), however, does not appear to be a risk factor as it was more common in the general population than among SARI patients, at least according to the national health survey.

The comparison of treatment procedures across the three pathogens showed broadly similar patterns. After adjusting for sex, age, comorbidities and nursing home residence, no significant differences were observed in length of hospital stay, ICU admission or treatment procedures. Other studies reported heterogeneous results: some identified longer hospital stays among patients with COVID-19 [19], others among patients with RSV [12-14] or influenza [15], while several found no difference [17,20]. Similarly, reported differences in ICU admission vary, with some studies describing higher admission rates among patients with influenza [13], others among patients with RSV [12,14-17] and others among patients with COVID-19 [19]. Both length of stay and ICU admission were influenced by factors beyond disease severity, such as hospital capacity constraints. This may have had a greater impact on patients with RSV and influenza, who are predominantly hospitalised during the busy winter season, than on patients with COVID-19.

Patients with RSV or influenza were more likely than patients with COVID-19 to experience a complicated disease course, primarily because pneumonia and acute renal insufficiency were the two most common complications. A key finding was the substantially higher case fatality rate among patients with RSV. After adjusting for sex, age, nursing home residence and comorbidities, this difference remained statistically significant between RSV and COVID-19, but it was no longer statistically significant for RSV vs influenza. The greater disease severity of RSV compared with influenza and COVID-19 has also been reported in several studies [14,16,18], whereas others have described a more severe course in COVID-19 (during the pandemic) [12], or influenza [13] or found no difference between infections [20].

A plausible explanation for the higher mortality among patients hospitalised with RSV is the difference in vaccination coverage between the three infections. During the study period, annual vaccination campaigns in Belgium reached adults aged 65 years and older and adults with comorbidities for both influenza and COVID-19, but not for RSV. Vaccination coverage for influenza in Belgium is ca 40–45% among adults with comorbidities and 55% among individuals aged 65 years and older [11,21,22]. For COVID-19, vaccination coverage is around 60% among adults with comorbidities and 40% among the older adults [23,24]. Both influenza and COVID-19 vaccinations have been shown to reduce not only the risk of hospitalisation but also the severity of disease in hospitalised patients [25-27]. Consequently, the higher case fatality rate and more frequent severe disease observed in hospitalised RSV patients may be attributable to lower vaccination coverage, rather than reflecting a more severe disease course inherent to the RSV virus itself. A study in the US found that RSV was similar in severity to COVID-19 or influenza among unvaccinated patients, but more severe compared to patients vaccinated for influenza or COVID-19 [28]. Because we did not have complete vaccination data for the patients, we could not adjust for vaccination status in our analysis or assess this hypothesis in our study. Also, the relatively higher proportion of nursing home residents and of patients older than 85 years could be a consequence of the higher vaccination coverage in nursing homes and by increasing age.

Some differences in disease course were observed between patients aged 18–64 years and those aged 65 years or older. Older patients had longer hospital stays but were admitted to intensive care less frequently, with ICU admission declining significantly with advancing age. This finding contrasts with the observation that complication rates did not decrease with age; in fact, complications such as cardiovascular events and acute renal insufficiency were more common in older patients. It also contrasts with the marked age-related increase in mortality. A possible explanation is that ICU admission and invasive procedures such as invasive ventilation may sometimes be considered inappropriate for older patients. The strong association between age and increased mortality has been consistently demonstrated in previous studies of COVID-19 [29-31], influenza [32,33] and RSV [34].

Although the risk profiles of patients were largely similar across all three pathogens, the vaccination recommendations of the Belgian SHC and the reimbursement criteria differ. For example, hypertension and cognitive disorders are not considered risk factors for severe influenza but are included for COVID-19, despite being equally prevalent in both patient groups. An important discrepancy concerns age-based recommendations: RSV vaccination is recommended by the SHC for all individuals 75 years and older, whereas the threshold is 65 years and older for both vaccination against influenza and COVID-19. Furthermore, RSV vaccination for adults with comorbidities is recommended only from the 60 years of age and reimbursed from 65 years of age, while influenza and COVID-19 vaccination is advised and reimbursed for all adults with comorbidities regardless of age. Although patients with RSV were, on average, older than patients with influenza, but not older than patients with COVID-19, the proportion of individuals aged 65–74 years was similar across the three groups: 23% for RSV, 24% for influenza and 22% for COVID-19. The difference becomes clear when comparing how many patients meet the current vaccination criteria. While 89% of influenza patients and 96% of COVID-19 patients in our study met the criteria for vaccination against their respective infections, only 81% of RSV patients did so. If we hypothetically apply the current criteria for influenza and COVID-19 vaccination to RSV vaccination, the proportion of SARI patients due to RSV that would have been eligible for vaccination increases to 94% and 96%, respectively. We acknowledge that factors such as infection incidence and vaccine cost also inform vaccination policy. Nevertheless, from an epidemiological and organisational perspective, harmonised vaccination criteria may offer stronger justification than infection-specific approaches.

Our study focused on only three pathogens. The rationale for this choice is that these are the main viral respiratory pathogens responsible for seasonal or periodic epidemics, which place a substantial burden on the healthcare system. This is also why they are the primary targets of vaccination campaigns. Consequently, our analysis did not include the large number of about 70% of SARI patients who tested negative for all three selected pathogens. Most of these cases are attributable to bacterial infections or infections with other viruses, although some may represent false-negative results for influenza virus, RSV or SARS-CoV-2, for example, due to sampling long after symptom onset.

Several limitations inherent to our study design should be acknowledged. First, we relied on routine hospital surveillance data, which may be subject to issues such as incomplete reporting or misclassification. However, following a comprehensive revision of the surveillance system in 2023, data quality improved substantially, and these data were considered sufficient for the analyses presented. Second, comparisons between pathogens could only be adjusted for variables routinely collected, such as age, sex, and nursing home residence; residual confounding from unmeasured factors cannot be excluded. Third, the summer months (May–September) were not included, as RSV testing is not routinely performed during this period. Consequently, the two COVID-19 waves of 2024, which occurred in summer, and RSV cases hospitalised early in the 2023/24 season (before 13 November) were not captured. Although it is theoretically possible that these patients may have differed in risk profile or disease course, we consider it unlikely that their inclusion would have substantially altered our findings.

A strength of our analysis is that we applied an improved case definition that better captures patients with RSV and COVID-19, whereas the World Health Organization SARI case definition is more focused on influenza and has a lower sensitivity for detecting influenza, RSV and COVID-19 [35,36].

## Conclusion

Our analysis shows that, over the past two seasons, patients hospitalised with RSV had a disease course comparable to, and in some cases more severe than, patients with influenza or COVID-19, with similar rates of ICU admission and complications and a higher case fatality rate. The introduction of new RSV vaccines therefore represents an important advance in prevention and public health. The risk profile for severe disease is highly comparable across the three pathogens, and the question can be asked if national recommendations for vaccination should not be more harmonised.

## Data Availability

Data will be made available on request to Sciensano (epirespi@sciensano.be).

## Supplementary Data

207000 Supplementary Material
