# Supplementary material for: Characteristics and outcomes of adult patients hospitalised with RSV infection, influenza or COVID-19 in Belgium, winter seasons 2023/24 and 2024/25
Source: Euro Surveill. 2026 Sep 17;31(37):2600088. doi: 10.2807/1560-7917.ES.2026.31.37.2600088 (PMC13589209; doi:10.2807/1560-7917.ES.2026.31.37.2600088)
Supplement: Supplementary file 1 [file 26-00088_LAFORT_Supplement.pdf]

This supplementary material is hosted by *Eurosurveillance* as supporting information alongside the article "Characteristics and outcomes of adult patients hospitalised with RSV infection, influenza or COVID-19 in Belgium, winter seasons 2023/24 and 2024/25", on behalf of the authors, who remain responsible for the accuracy and appropriateness of the content. The same standards for ethics, copyright, attributions and permissions as for the article apply. Supplements are not edited by *Eurosurveillance* and the journal is not responsible for the maintenance of any links or email addresses provided therein.

Supplementary table S1: Sociodemographic characteristics and risk factors of patients 18-64 years and 65 years and older hospitalised for respiratory syncytial virus, influenza or COVID-19, by sub-age category, Belgium, winter seasons 2023/24 (13 November 2023–30 April 2024) and 2024/25 (1 September 2024–30 April 2025)

	18-54 years	55-64 years	p-value	65-74 years	75-84 years	85+ years	p-value
Socio-demographic characteristics							
Female	50.8%	54.1%	0.362	42.4%	45.5%	51.1%	**0.007**
Nursing home resident	1.7%	2.1%	0.689	5.5%	12.1%	32.9%	**<0.001**
Comorbidities							
At least one	76.2%	87.3%	**<0.001**	95.4%	95.7%	96.3%	0.751
Asthma	18.0%	14.5%	0.189	10.2%	12.6%	8.8%	0.093
Other chronic respiratory disease	17.6%	39.0%	**<0.001**	47.0%	33.2%	20.2%	**<0.001**
Chronic cardiac disease	10.9%	27.4%	**<0.001**	40.2%	80.8%	58.9%	**<0.001**
Hypertension	12.1%	27.9%	**<0.001**	39.5%	47.1%	49.2%	**0.002**
Diabetes	10.9%	19.0%	**0.002**	29.5%	28.0%	23.1%	**0.033**
Obesity	9.7%	12.7%	0.209	15.7%	13.6%	6.8%	**<0.001**
Liver insufficiency	3.4%	7.0%	**0.025**	5.5%	3.3%	2.5%	**0.021**
Renal insufficiency	9.3%	13.3%	0.089	19.2%	27.8%	35.7%	**<0.001**
Immunodeficiency	18.4%	21.6%	0.265	17.7%	14.1%	3.6%	**<0.001**
Organ transplant	3.3%	3.8%	0.716	2.3%	0.9%	0.0%	**0.001**
Stem cell transplant	3.2%	4.0%	0.600	4.6%	0.0%	0.0%	**<0.001**
Solid cancer	5.7%	18.4%	**<0.001**	22.6%	25.9%	18.3%	**0.006**
Haematologic cancer	8.5%	9.2%	0.723	13.2%	9.2%	2.9%	**<0.001**
Other haematologic disease	0.9%	4.9%	**0.012**	4.5%	8.1%	6.4%	0.164
Neurologic disease	8.9%	10.0%	0.609	10.6%	15.3%	19.5%	**<0.001**
Neuromuscular disease	5.2%	4.1%	0.487	2.0%	3.5%	3.9%	0.135
Cognitive disturbance	5.7%	4.3%	0.426	6.6%	18.0%	26.1%	**<0.001**

Statistically significant associations ($p < 0.05$) in bold
